# Inference of the infection status of individuals using longitudinal testing data from cryptic populations: Towards a probabilistic approach to diagnosis

**DOI:** 10.1038/s41598-017-00806-4

**Published:** 2017-04-19

**Authors:** Svetlana N. Buzdugan, Timothée Vergne, Vladimir Grosbois, Richard J. Delahay, Julian A. Drewe

**Affiliations:** 1 Royal Veterinary College, London, UK; 2 Centre de Coopération Internationale en Recherche Agronomique pour le Développement, Montpellier, France; 3 National Wildlife Management Centre, Animal and Plant Health Agency, Woodchester Park, Gloucestershire, UK

## Abstract

Effective control of many diseases requires the accurate detection of infected individuals. Confidently ascertaining whether an individual is infected can be challenging when diagnostic tests are imperfect and when some individuals go for long periods of time without being observed or sampled. Here, we use a multi-event capture-recapture approach to model imperfect observations of true epidemiological states. We describe a method for interpreting potentially disparate results from individuals sampled multiple times over an extended period, using empirical data from a wild badger population naturally infected with *Mycobacterium bovis* as an example. We examine the effect of sex, capture history and current and historical diagnostic test results on the probability of being truly infected, given any combination of diagnostic test results. In doing so, we move diagnosis away from the traditional binary classification of apparently infected versus uninfected to a probability-based interpretation which is updated each time an individual is re-sampled. Our findings identified temporal variation in infection status and suggest that capture probability is influenced by year, season and infection status. This novel approach to combining ecological and epidemiological data may aid disease management decision-making by providing a framework for the integration of multiple diagnostic test data with other information.

## Introduction

Detecting infection with confidence in individuals can be difficult when diagnostic tests are imperfect because the true state of infection is not, or is only partially, determinable^1^. Cryptic populations of wild animals add an extra layer of complexity because they are often unobserved for long periods and may be difficult to catch and sample. This poses a problem because infectious diseases in cryptic populations can be immensely important: the majority of emerging infectious diseases originate in wildlife^2, 3^. Methods for detecting infection in such populations need to try to account for hidden or uncertain processes if they are to reveal the true dynamics of disease and help us to develop effective disease control interventions.

Capture-recapture (CRC) methods were originally developed to gain insights into human demographic data^4^, and have since been used extensively in the ecological sciences to estimate demographic parameters in wild animal populations^5^. Such methods use information gleaned from sequences of detections and non-detections to estimate the number of missing individuals and hence allow adjustment for undercounting in population surveys^6, 7^. In epidemiology, the use of CRC was initially limited to estimating the number of infected individuals that were unrecorded (‘missed cases’), an application which has been popular for more than 30 years in the study of chronic and infectious diseases in humans^8^ and, more recently, in livestock^9^. The use of CRC to estimate disease transmission parameters to gain insights into the dynamics of infectious diseases is a more recent development^10–13^.

The potential effects of heterogeneity in state-specific detection probabilities on estimates of disease prevalence were demonstrated by Jennelle *et al*.^14^ in a study of *Mycoplasma gallisepticum* infection in house finches (*Carpodacus mexicanus*). In this system, detection probabilities of uninfected finches were generally higher than of infected individuals^14^. Rossi *et al*.^12^ used a multi-state CRC model^15^ to estimate rates of infection and immunity in relation to classical swine fever virus in wild boar. Diagnostic inaccuracy due to imperfect test sensitivity and specificity was not addressed, creating an unresolved problem when individuals tested negative (uninfected or susceptible) after previously testing positive (infected) because in real life animals infected with classical swine fever virus are known to either die or recover with immunity, but never to revert to a susceptible state^12^. Lachish *et al*.^11^ examined the impact of devil facial tumour disease in Tasmanian devils on age- and sex- specific apparent survival rates as well as variation in the rate of transition from healthy to diseased state. Misclassification of disease status occurred because diagnosis was based solely on the observation of clinical signs, leading to limited sensitivity due to a long latent period when infected individuals show no signs of disease^11^. These examples of infection state misclassification are sources of bias that may impact considerably on the interpretation of disease dynamics and the success of management interventions.

The problem of imperfect state observation (which includes false positive and false negative diagnostic test results) was discussed by Conn and Cooch^10^. They used a multi-event CRC modelling framework to incorporate data from uncertain or unknown states, a process they claimed increased the precision of disease dynamic parameter estimates^10^. Although such a method may help deal with uncertainty in ascertaining state, it doesn’t deal with misclassification bias because of the implicit assumption that the state of an individual (e.g. its infection status) is determined accurately if information on state is obtained at all^10^. This bias remained unaddressed in a recent study of host–pathogen dynamics in rabbit populations^13^ although the authors claim its impact was likely to have been negligible due to diagnostic test sensitivity and specificity both being at least 98%. However, diagnostic tests for many problematic infectious diseases – such as tuberculosis in cattle and badgers^16, 17^ – are less accurate, and hence the problem of imperfect state observation needs addressing.

One potential way to improve diagnostic accuracy is to use multiple tests in series or parallel^18^. For example, combined use of three different diagnostic tests for tuberculosis in live badgers increases detection of infection with *Mycobacterium bovis* ^19^, particularly when interpreted at the social group level^20^. A potentially major limitation of such analyses, however, is that a diagnosis is frequently based on just one sampling event. Hence any previous test results from the same individual are not accounted for in the diagnosis. This wastes potentially useful information for inferring current infection status in individuals that are repeatedly sampled over time (for example, those with a chronic or recurring infection), particularly given the imperfect performance of diagnostic tests.

Although imposing a binary classification of disease status – where an individual is considered to be either infected or not – may be the norm, it is problematic when information on state is imperfect. One possible solution is to consider the move from uninfected to infected as a continuum such that an individual can be partially infected, perhaps based on a spectrum of immune responses^21^. This is not entirely satisfactory, however, and a more intuitive approach is to consider that state may be discrete but our certainty about it is not. Hence, a probability is associated with an individual’s infection status at any given point in time, depending on prior and current knowledge.

In the present study we use data from a longitudinal field study of wild badgers in south-west England to explore the challenges described above. Empirical data from this cryptic population includes the results of three different imperfect diagnostic tests for *M. bovis* infection, accumulated during sequential captures of individually-marked badgers over many years. Within this dataset it is not uncommon to find test-positive individuals subsequently testing negative (using the same diagnostic test) at the next sampling event^19^. Graham *et al*.^22^ incorporated prior test result information from these animals using multi-state capture-recapture models^15^, although they did not account for imperfect diagnosis arising from the combined use of a serological and a culture test^19^. In the current study, we use a multi-event capture-recapture approach^23^ to model imperfect observations of true epidemiological states. Our objective is to estimate the probability that any individual badger is truly infected using information from the whole of its capture history and any combination of previous diagnostic test results. Of course, we do not usually know an individual’s true infection status: rather, we have a set of diagnostic test results and from these we wish to infer the probability of an individual being infected. Here, we show how to do this in an approach where the parameters reflecting the capture, testing, and epidemiological state transition processes are first estimated by fitting multi-event capture-recapture models to capture and testing history and then combined to produce estimates of the probability of a badger being infected given its capture and testing history.

The inclusion of historic sampling patterns and previous diagnostic test results in the interpretation of current diagnostic test results would be expected to reduce the uncertainty on the true infection status of individuals. Such information could then be used to improve the reliability of decisions made to manage disease in populations where diagnostic test results are available from the same animals over a period of time. This could provide new insights into infectious disease dynamics, particularly in cryptic populations.

## Results

From July 2006 to October 2013, 541 different badgers were captured and sampled during 28 capture sessions. Badgers were often caught multiple times (median: 3, range: 1 to 18 times per badger) giving a total of 2,022 sampling events during the study period. At every sampling event, three tests (StatPak, interferon-gamma assay, and culture) were performed on samples collected from each badger. All eight possible combinations of test results were observed (Table 1) because each of the diagnostic tests was imperfect, and because each test targeted a different measure of infection or immune response and therefore was potentially identifying animals at different stages of disease progression.

**Table 1:** Observed frequencies of the eight possible combinations of results from three diagnostic tests used in 2,022 sampling events involving 541 different badgers.

	Test 1−		Test 1+	
	Test 2−	Test 2+	Test 2−	Test 2+
Test 3−	1437 [1]	164 [3]	208 [2]	165 [5]
Test 3+	3 [4]	3 [7]	6 [6]	36 [8]

Numbers in square brackets identify each of the possible test result combinations referred to in Fig. 5.

Test 1 = StatPak; Test 2= interferon-gamma assay; Test 3 = Culture; += positive test result; −=negative test result.

The model presenting the best compromise between its goodness of fit and its complexity indicated that the capture probability varied significantly according to badger sex and infection status, season, year, and the interaction between sex and season. The probability of transition from the uninfected to the infected state was shown to be influenced only by season and year. The probability of being infected at first capture was only dependent on the year of first capture. Parameters associated with the first year (2006) and the last year (2013) for recapture probability and transition probability were estimated at the boundary with extremely large confidence intervals. This was most likely due to lack of data for the first year and identifiability issues for the last year, both of which are common problems in time-dependent models. Such models require substantial amounts of data, which means it is often not possible to separately estimate parameters referring to the last time step of a survey (e.g. the last transition and recapture parameters). Rather, only quantities that combine them, such as products of the parameters, can be estimated^24, 25^. Consequently, results hereafter refer to the five-year period between 2007 and 2012.

### Re-capture probability

**Figure 1:**
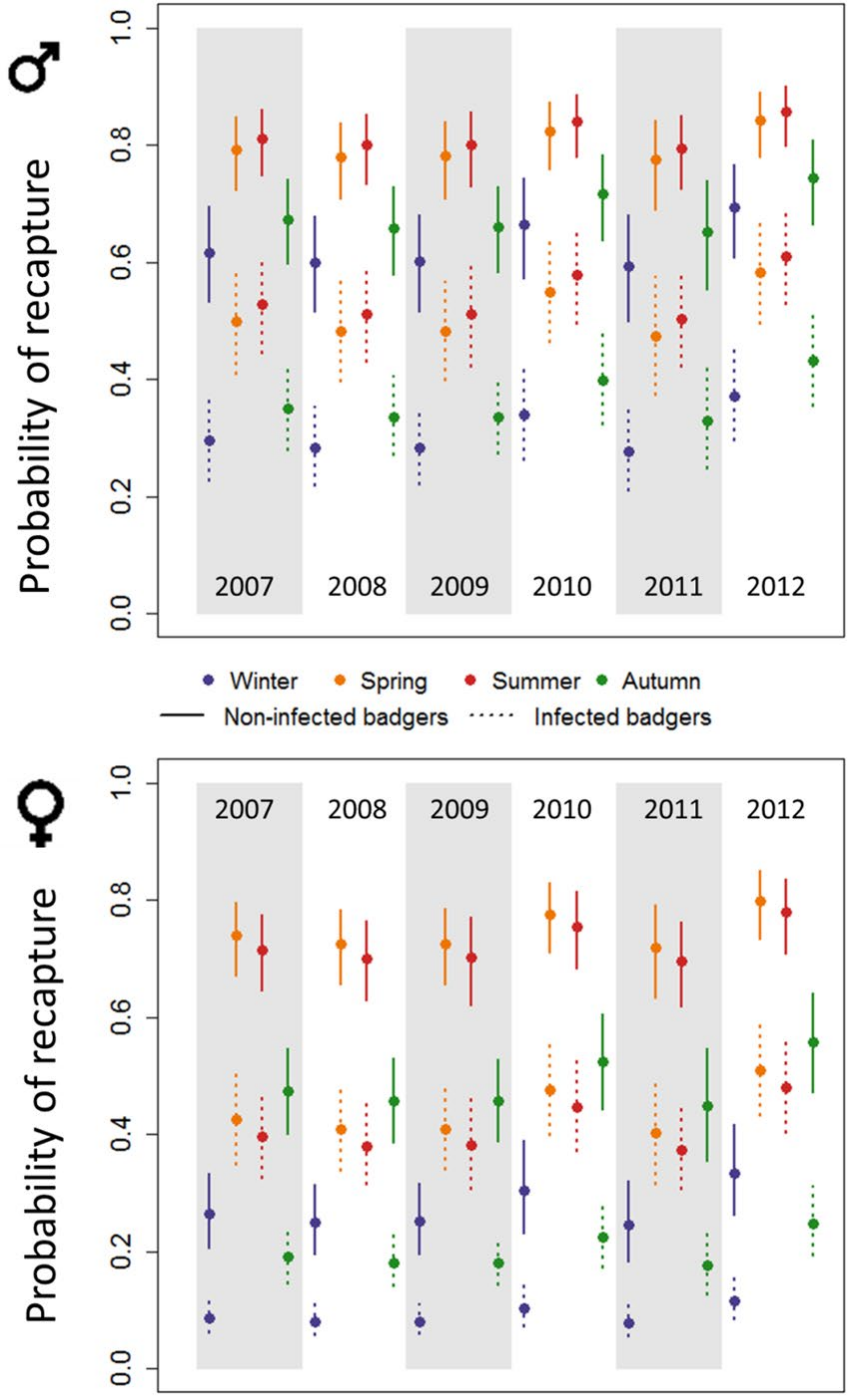
Temporal dynamics of the recapture probability for uninfected (solid lines) and infected (dotted lines) badgers at Woodchester Park from 2007 to 2012. Upper graph: males. Lower graph: females. Circles and bars indicate point estimates and 95% confidence intervals, respectively.

Re-capture probability was systematically higher for males than females (odds ratio [OR] = 1.70; 95% confidence interval [CI] = 1.23 to 2.38). Infected badgers were far less likely to be re-captured than were uninfected badgers (OR = 0.26; 95% CI: 0.20 to 0.34). Significant seasonal and annual variations were also detected, with re-capture probabilities increasing in spring and summer (Fig. 1). The significant interaction between sex and season highlighted greater seasonal variation in re-capture probability amongst females than males, with a marked reduction in re-capture probability of females in winter. Overall, the probability of re-capturing a male badger, given that the badger was alive and available to be caught, varied between 0.2 and 0.9 (depending on its epidemiological state, the year and the season) whilst for females it varied between 0.05 and 0.6 (Fig. 1).

### Dynamics of transmission of *M. bovis* infection among badgers

The probability of being infected at first capture was associated only with the year of capture (ranging from 0.06 (95% CI: 0.02 to 0.18) in 2007 to 0.40 (0.20 to 0.64) in 2011: Supplementary Table S1). Note that at every capture session after the first one, a portion of badgers had been sampled previously, and hence the population that could be captured for the first time was not representative of the badger population. This means that the probability of being infected at first capture is not an accurate reflection of the prevalence of infection in this population.

**Figure 2:**
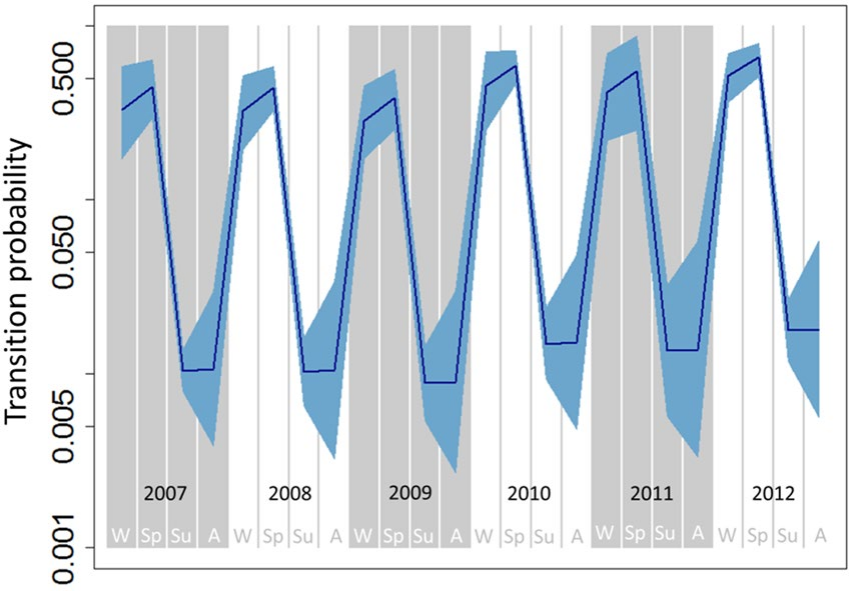
Temporal dynamic of the probability that an uninfected badger becomes infected between two capture sessions. Blue shading indicates 95% confidence intervals. The y-axis is on the log-scale. W: winter; Sp: spring; Su: summer; A: Autumn.

The probability of transition from the uninfected to the infected state between capture sessions varied with season and year (Fig. 2). While the probability of becoming infected between capture sessions in either summer or autumn and the following session was negligible, it varied between 5% and 39% during winter and spring. The spring seasons of 2010, 2011 and 2012 were associated with the highest transition probabilities of the whole study period (30%, 25% and 39%, respectively), indicating that about one quarter to one third of the uninfected badgers in the population at that period were likely to become infected. The sex of the badger was not found to be significantly associated with transition probability.

### Probability of observing the test results given infection status

The probabilities of obtaining each of the eight possible combinations of test results were estimated as functions of the infection status of each captured badger by fitting the multi-event model to the capture and test result histories of each individual (Table 2). The most likely test result combination for an *uninfected* badger was for all three tests to be negative (combination 1), with a probability of 0.94 (95% CI 0.91 to 0.96). However, for *infected* badgers, the probability of testing negative on all three tests (0.27 [95% CI 0.22 to 0.33]) was the same as the probability of testing positive on just one test (combination 2: StatPak, probability = 0.27 [95% CI 0.23 to 0.31]) and similar to the probability of testing positive on two tests (combination 5: StatPak and the interferon-gamma assay, probability = 0.23 [95% CI 0.20, 0.27]).

**Table 2:** Probability of observing each combination of diagnostic test results given the true epidemiological status as uninfected or infected.

Combination of test results	Test 1 (StatPak)	Test 2 (inter-feron-gamma assay)	Test 3 (Culture)	Probability of the combination of test results			
				If uninfected		If infected	
				point estimate	95% CI	point estimate	95% CI
1	−	−	−	0.94	0.91, 0.96	0.27	0.22, 0.33
2	+	−	−	0.02	0.01, 0.03	0.27	0.23, 0.31
3	−	+	−	0.04	0.03, 0.06	0.16	0.13, 0.20
4	−	−	+	<0.01	−	<0.01	−
5	+	+	−	<0.01	−	0.23	0.20, 0.27
6	+	−	+	<0.01	−	0.01	0.00, 0.02
7	−	+	+	<0.01	−	<0.01	−
8	+	+	+	<0.01	−	0.05	0.04, 0.06

CI = confidence interval; + = positive test result; −= negative test result.

It should be noted that the sensitivity and specificity of the three diagnostic tests are not set to known values in the multi-event capture-recapture model. Instead, the multi-event capture-recapture model includes parameters related to the sensitivity and specificity of the diagnostic tests and which are estimated (and not set to any previously known values). These parameters are the 16 probabilities of observing a given test result combination given the true epidemiological status of the badger (the eight combinations presented in Table 1 for both infected and uninfected animals). The estimations produced by the multi-event capture-recapture model for these parameters are given in Table 2, and may be used to derive estimates for the sensitivity and specificity of each diagnostic test. For instance, an estimation of the sensitivity of the StatPak test (the first test in the notation of the test results combination) can be obtained by summing the estimations of P(+ + +|inf), P(+ − +|inf), P(+ + −|inf) and P(+−|inf) in Table 2. Likewise, the specificity of the StatPak test can be obtained by summing the estimations of P(−|uninf), P(−+|uninf), P(− + −|uninf) and P(− + +|uninf). By doing so, one obtains for sensitivities: 0.56 for StatPak, 0.44 for the interferon gamma assay, and 0.06 for culture. And for specificities: 0.98 for StatPak, 0.96 for the interferon gamma assay, and 1.00 for culture.

### Probability of infection status given observations

**Figure 3:**
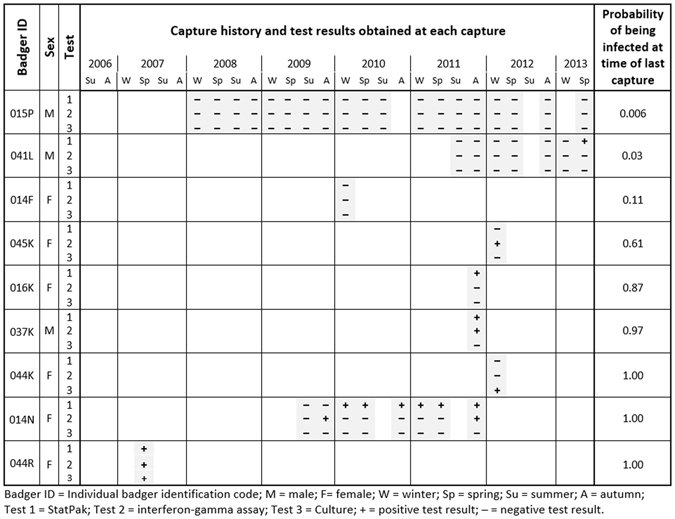
Examples showing variation in the probability of being infected with *M. bovis* at the time of last capture for a selected sample of badgers, given each individual’s history of capture and test results. The probability of a badger being truly infected varied with its sex, capture history and current and historical diagnostic test results. From July 2006 to October 2013 there were 28 time steps (seasons) when trapping and sampling of badgers occurred. A set of three diagnostic test results appears on a badger’s timeline if it was captured and tested at that time step. Hence badger 015P (top row) was caught and sampled 19 times during the study and on every one of these occasions it tested negative on all three tests. Our model estimated that the probability of it being truly infected at the time of its last capture in 2013 was 0.006 (indicating that despite consistently testing negative over a long time period, the insensitivity of the testing regime and the background prevalence of infection in the population meant this badger nevertheless had about a one in 170 chance of being infected). At the other end of the scale, high probabilities of infection can result from a single positive result if that test has very high specificity (e.g., badger 044 K, which tested positive for *M. bovis* using the highly-specific culture test) or from repeated positive results to less specific tests (e.g., badger 014N). The probabilities shown were estimated using a multi-state capture-recapture model described in the main text.

Notwithstanding the above, an individual’s true infection status is usually unknown. Rather, we have a set of diagnostic test results from which we wish to infer the true infection status of the individual. This can be achieved by combining the individual capture histories with the probabilities of capture, being infected at first capture, becoming infected (transitioning from uninfected to infected) and of obtaining each combination of diagnostic test results, as estimated from the multi-event capture-recapture model. Figure 3 shows the capture and diagnostic test result histories for a selection of badgers, and illustrates the effect of sex, capture history and current and historical diagnostic test results on the probability of being truly infected given any diagnostic test result. For example, a female that was sampled only once in winter 2010 and tested negative on all three tests (see badger 014F in Fig. 3) would nonetheless have a 11% probability of in reality being infected but undetected owing to the insensitivity of the tests. Obtaining one or more positive test results increased the probability that a badger was truly infected at its most recent capture (see badgers 016 K, 037 K and 044 K in Fig. 3). In contrast, badgers that were caught many times over several years and consistently tested negative were unlikely to be truly infected, although this probability never reached zero (see badger 015P in Fig. 3); this is likely due to the imperfect sensitivity of the testing regime and the background prevalence of infection in the population - which was estimated in a recent paper to have reached 30% in 2005, just prior to the start of this study^26^.

**Figure 4:**
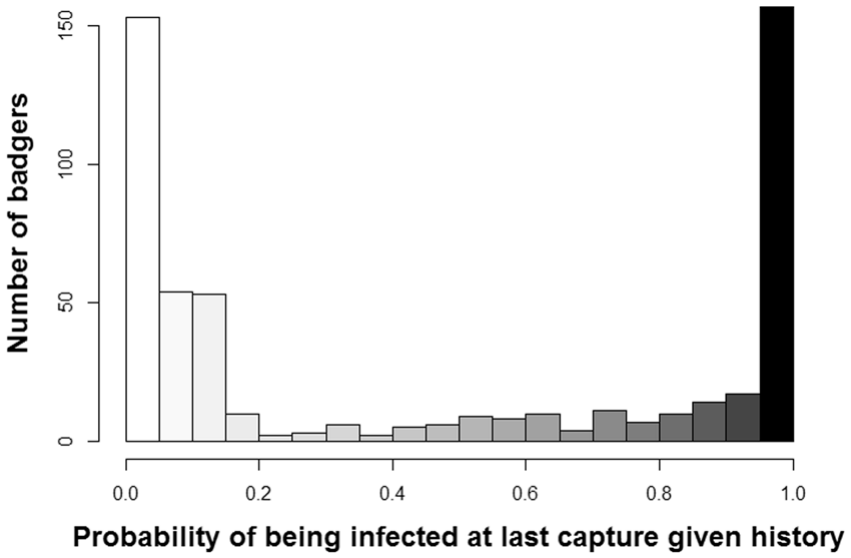
Distribution of the probability of being infected in the Woodchester Park badger population at the time of last capture of each individual, given its capture and testing history. Approximately 70% of subjects in the study population had probabilities of being infected at their last capture that were higher than 0.95 (black bar) or lower than 0.05 (white bar), meaning that the infection status of the remaining 30% of badgers was less certain. Note that the 5% and 95% thresholds are used here arbitrarily: any cut-off could be selected depending on the degree of certainty required.

Whilst 38% of captured badgers had less than 5% probability of being truly infected at their most recent sampling event, 32% of captured badgers had more than 95% probability of being truly infected (Fig. 4). Consequently, 30% of badgers were in an epidemiological state that could not be ascertained with more than 90% certainty. This is demonstrated in Fig. 3 by badgers 045 K and 016 K, which were estimated to have a 61% and an 87% probability of being infected, respectively. Both of these individuals were sampled only once and each animal generated a positive result on a (different) single test. Badger 045 K had a notably lower probability of infection than did badger 016 K which is likely to be at least in part because of the difference in season when each animals was first caught; the probability of being infected at first capture in winter 2012 (badger 045 K) was lower than that for animals first caught in autumn 2011 (badger 016 K). Since the estimates for the probability of infection were updated each time an individual was caught and sampled, certainty about infection status tended to increase for animals caught on more than one occasion (Fig. 3).

## Discussion

In this study we used a unique long-term empirical dataset of badger capture and testing histories to estimate the probability of infection with *M. bovis*, for each individual badger, given any combination of results from three imperfect diagnostic tests, and accounting for each animal’s capture and test result history. In doing so, we reduced the impact of bias associated with imperfect diagnostic test performance and cross-sectional sampling, by expressing the likelihood of infection at an individual level rather than categorising an animal as either infected or uninfected. Although such an approach does not remove the need for interpretation in order to make decisions on how to manage infection in an individual (or population), it does create opportunities to vary the level of confidence associated with such decisions depending on the circumstances. The desired threshold of confidence will depend on the implications of making the wrong decision (e.g. of mistakenly removing an individual which is in fact uninfected). For long-term surveillance of disease in this population, applying a probabilistic approach to infection status represents a clear improvement on current practice where a positive or negative diagnosis is usually based on imperfect tests applied at a single sampling point^26, 27^. Our findings suggest it is possible to gain a much deeper insight into the true infection status of individual badgers by interpreting longitudinal diagnostic test results and accounting for uncertainty in biological and behavioural processes. In the present study, these processes included the probabilities of badgers being captured and becoming infected, both of which varied with season and year. We suggest that such sources of uncertainty should not be ignored when attempting to evaluate population infection dynamics, particularly in cryptic wild animal populations.

Capture probability was strongly associated with infection status, such that infected badgers were four times less likely to be re-captured than were uninfected badgers. A higher probability of detecting uninfected (compared to infected) individuals has also been shown in house finches infected by *Mycoplasma gallisepticum* ^14^. There are several important potential consequences of this finding. When the detection probability of uninfected individuals is higher than infected individuals, apparent prevalence will be negatively biased. In the present study, the heterogeneity in detection of infected versus uninfected individuals provides further support to the suggestion that there may be behavioural consequences of infection. In previous studies, *M. bovis* infection in badgers has been associated with differences in ranging behaviour^28^, likelihood of visits to farm buildings^29^, sett use and social network position^30^. However, none of these studies were able to ascertain whether infection affected behaviour or vice versa. The results of the present study suggest that infection may affect subsequent behaviour, because it is hard to imagine how the act of being captured could influence infection risk. The proposition that infected badgers may be more difficult to capture in cage traps would clearly have significant implications for disease control interventions that employed this approach. In the case of cage trapping to cull, it would mean that uninfected animals were preferentially removed, which would reduce the effectiveness of the operation. This phenomenon could potentially have contributed to the increase in *M. bovis* prevalence observed over sequential culls during the Randomised Badger Culling Trial^31^. By contrast, in the case of cage trapping to vaccinate, the preferential capture of uninfected animals would be expected to increase cost-effectiveness as vaccination does not appear to have any therapeutic effect in infected individuals^32, 33^. These findings could also be significant for other research studies using cage trapping, as ignoring differential capture rates for infected and uninfected individuals would result in biased estimation of epidemiological parameters. However, the possibility that infected animals may be less likely to be cage-trapped warrants further investigation before we can determine whether it is a substantial and widely applicable phenomenon.

Our method of data collection was based on trapping and sampling; the amount of movement of badgers between the three data collection zones was not measured. The vast majority of badgers (98.5%) were trapped in only one of the three zones within a season. While it is possible that some of these badgers may have travelled to (but not been trapped in) other zones, this will not have affected the study’s findings or our interpretation of results because the model explored the emergent properties of the whole system rather than speculating on the possible mechanisms that underlie it. In the present study, badgers were much more likely to be caught in spring and summer than in winter. This corroborates previous findings that the probability of capturing badgers varies by season^22, 34^. This is likely to arise owing to fluctuations in weather conditions affecting food availability and foraging behaviour^35, 36^ in addition to cubs becoming independent (and therefore more readily trappable) in the summer^37^. Trapping effort during the long term study at Woodchester Park has remained constant from one year to the next^26^ and therefore should not have influenced these estimates. Our model output indicated very clear seasonality of *M*. *bovis* transmission in badgers, with most transfer from an uninfected to an infected state occurring during winter and spring, and very little in the summer and autumn. It is possible that this pattern could be related to increased social contact associated with breeding and the birth of cubs, or to the seasonal aggregation of social group members at the main sett which might lead to increased transmission through shared airspace, grooming or aggression, but further research would be needed to test these hypotheses.

There is a high level of uncertainty associated with conventional methods of interpreting diagnostic test results in live badgers, due to the tests’ inherent insensitivity and imperfect specificity^20^. Current methods of interpretation often assume that a badger is infected from the point at which it produces its first positive diagnostic test result, although this is likely to miss several cases of infection due to limitations in test sensitivity. Diagnostic accuracy can be improved by interpreting several tests together^19^ and can be further improved by also incorporating information gained from previous testing of the same animal. The results of the present study indicate that the probability of a badger being identified as truly infected depends upon, among other things, its sex, the duration of follow-up, gaps in capture history (as infected badgers are less likely to be captured), the availability of previous test results and the probability that the tests will correctly give a positive or negative diagnosis. It therefore makes sense to incorporate as much of this information as possible into the diagnostic process. Our results suggest that the likely infection status of a badger that tested negative a long time ago and hasn’t been caught since should perhaps be considered differently to a badger that has recently been caught and tested negative: the former will have a higher probability of being infected according to our model, as not all negative test results are equal! Hence, when considering data from the long term study at Woodchester Park, although it might seem reasonable to consider that a badger trapped several times and always yielding a negative test result was likely to be uninfected, our new approach indicates that in many cases there would be a high likelihood that it was infected (especially if it had not been sampled for a protracted period).

This novel approach to considering ecological and epidemiological data in combination may aid decision-making in the management of disease in a wide range of species by providing a framework for diagnostic test data to be integrated with other information. Our method generates an infection probability value for each individual, so different thresholds for intervention (e.g. removal of an animal from the population) could be set according to the circumstances. An example of operational use would be as part of the exit from a management strategy which had been successful in reducing the prevalence of infection in the target population, and hence where a high level of confidence was required to remove the relatively low number of remaining infected animals. The approach would be of even more value where further removal of uninfected animals would be highly undesirable, as would be the case following any vaccination campaign. In the case of managing TB in badgers, both conditions would apply during an exit strategy at the end of a test, vaccinate or remove (TVR) campaign. As a research tool, the probability-based approach described here provides better prediction of infection status at the individual and population levels and hence produces more confidence in the outputs of the analysis of epidemiological data. Our method could be adapted to include other test results, for example environmental samples^38^. Incorporation of test results at the badger social group level^20^ could also be a useful refinement to account for the spatial aggregation of infection observed in this population^27^. Possible wider applications might include the interpretation of results from regular testing of farm animals for diseases affecting production^9^, and the results of testing humans for high-impact diseases^39^.

Our model gives a different interpretation for each possible combination of diagnostic test results. In doing so, we move diagnosis away from the traditional binary classification of infection status (infected versus uninfected) towards a probabilistic interpretation where the probabilities are updated each time the subject is re-tested. This encourages us to change the way we think about what it means to be infected. It may take a little time to comprehend what it means to say that a subject has a ‘90% chance of being infected’ but this interpretation is inherently more useful than a simple binary diagnostic classification because it acknowledges and quantifies the small chance that the subject may in fact be uninfected. Our results suggested that about 70% of badgers in the study population could be assigned as being either infected or uninfected with at least 90% confidence, meaning that the infection status of the remaining 30% of badgers was less certain. Previous methods of interpretation would have assigned these latter individuals as either infected or uninfected. Hence our model allows us to accommodate rather than ignore uncertainty and account for the possibility of false positive and false negative test results.

In conclusion, we have demonstrated a multidimensional approach for inferring individual-level probabilities of infection from longitudinal testing data in a cryptic population. We have applied it to the interpretation of diagnostic test results for *M. bovis* infection in live badgers, where not every individual is available for testing and the available diagnostic tools are imperfect. This method has enabled us to uncover some of the underlying biological complexities influencing several epidemiological parameters, as well as our measurement of these parameters, and paves the way for the application of similar models to better understand the epidemiology of other diseases in species where longitudinal data are currently underexploited.

## Methods

### Ethics statement

Badger capture and sampling was carried out in accordance with licences from Natural England and the UK Home Office. The protocols were approved by local ethical review within the Food and Environment Research Agency and the Animal Health and Veterinary Laboratories Agency (now the Animal and Plant Health Agency).

### Study area and data collection

Data originated from fieldwork undertaken from July 2006 to October 2013 at Woodchester Park, Gloucestershire, UK (51° 43*′* N, 2° 16 W). This 7 km^2^ study area is home to a population of wild badgers that has been the subject of long-term research into badger ecology and TB epidemiology^26^. For trapping purposes, the study area was divided into three zones of approximately equal size, each of which was usually subject to four seasonal trapping sessions per year (Supplementary Table S2). Each trapping session lasted for three consecutive nights: in the rare instances where the same badger was captured twice during the same capture session, data from only the first capture was included in the analysis. Data from all three trapping zones were aggregated into one during each season, since badgers rarely were trapped in more than one zone (between July 2006 and October 2013, eight of 541 trapped individuals (1.5%) were trapped in more than one zone within a season). Trapping was suspended from February to April inclusive each year to avoid the capture of new-born cubs and their separation from lactating females.

Badgers were captured in the immediate vicinity of their setts in peanut-baited cage traps and transported to a sampling facility to be anaesthetised and sampled^26^. On first capture each badger was sexed and given a unique identifying tattoo on its ventral abdomen^40^ which allowed subsequent identification. Samples of blood, oesophageal and tracheal aspirate, urine, faeces, and swabs of any wounds were collected from each badger at every capture event. After recovery, all badgers were released at the point of capture.

Three independent diagnostic tests for *M. bovis* infection were performed on the samples collected. Test 1 was a commercially-available immunoassay (BrockTB StatPak; Chembio Diagnostic Systems, New York, USA) used to examine serum for IgM and IgG antibodies to the *M. tuberculosis*–complex antigens MPB83, ESAT-6 and CFP10^41, 42^. Test 2 was an interferon-gamma assay of cell-mediated immunity based on the stimulation of lymphocytes in whole-blood culture and the subsequent detection of interferon-gamma by sandwich ELISA^43^. Test 3 was culture of all samples except blood for mycobacteria using standard techniques^44^. Full details of all tests appear in Drewe *et al*.^19^. The capture history of each badger was summarised from the first to the last capture session as a series of numbers from zero to eight, where zero indicated that it was not captured on that occasion, and values from 1 to 8 corresponded to the eight possible combinations of diagnostic test results (Table 1).

### Multi-event capture-recapture model

A multi-event capture-recapture model^23^ was fitted to the whole dataset of capture histories, with infection status as the latent variable. Briefly, it was considered that at first capture, each badger could be in one of two epidemiological states: uninfected [susceptible] or infected. It was further assumed that transition from the uninfected to the infected state could occur between any successive capture sessions. However, consistent with the epidemiology of *M. bovis* in badgers, the model did not allow any reversion of the epidemiological state from infected to uninfected^27^. Given the true epidemiological state of a badger that was captured, each combination of diagnostic test results could be observed with estimable probabilities depending on the sensitivity and the specificity of each test (Table 2 and Supplementary Table S3).

**Figure 5:**
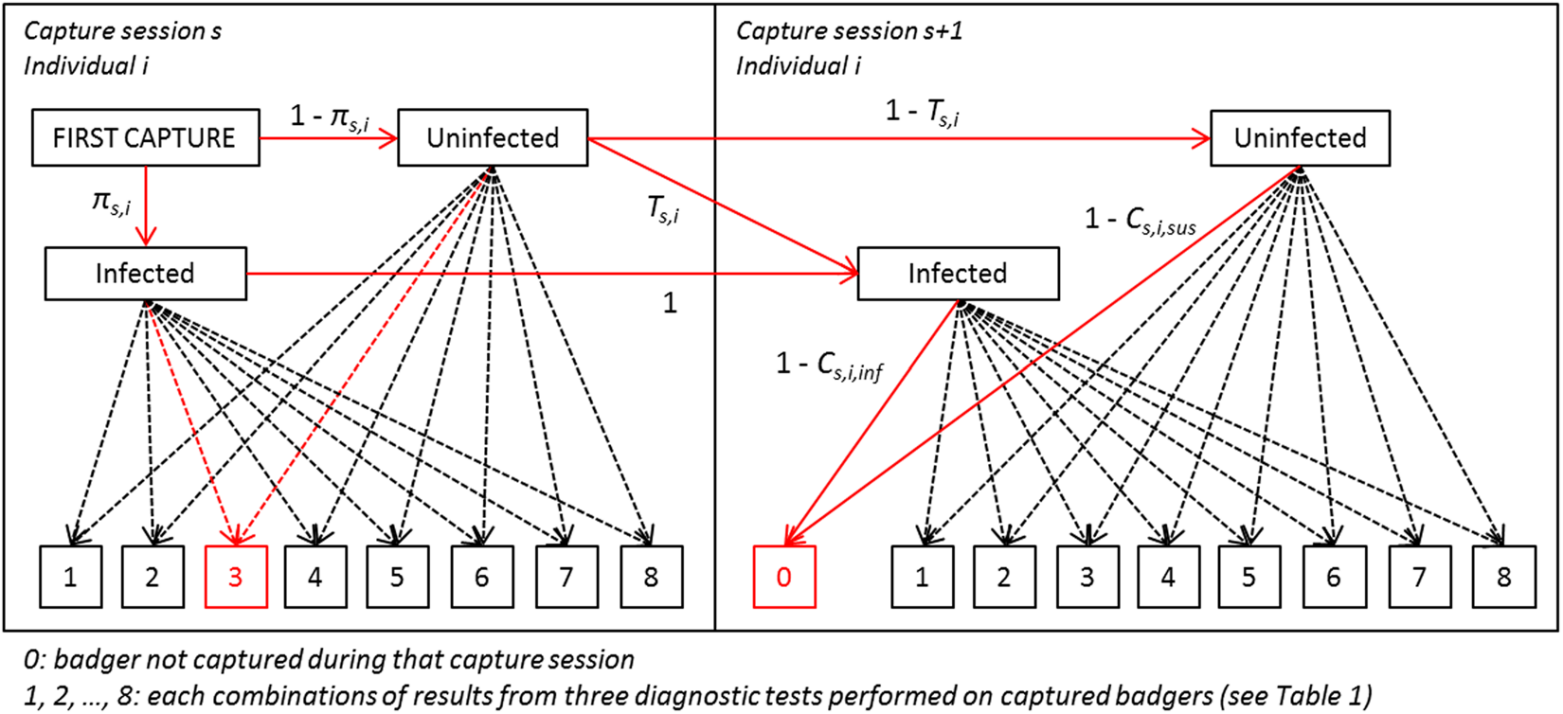
Multinomial tree diagram of the probability structure of the model at first and second capture. π_s,i_ is the probability of being infected at first capture at capture session s (characterised by the external variables year and season) for individual i (characterised by the external variable sex). T_s,i_ is the probability of transitioning from the uninfected to the infected state between capture sessions s and s + 1 for individual i. Finally, C_s,i,inf_ and C_s,i,sus_ are the capture probabilities at capture session s for individual I, for infected and uninfected badgers, respectively. Probabilities associated with the dashed arrows can be expressed as the product of the corresponding capture probability (C_s,i,inf_ or C_s,i,sus_) and the probability of observing the associated diagnostic test result combination given the epidemiological state (Supplementary Table S3). The red arrows highlight all pathways that could lead to the observation (3, 0): (the badger was infected at its first capture and the diagnostic test results showed combination 3 AND the badger was still infected at the following capture session when it was not captured) OR (the badger was not infected at its first capture and the diagnostic test results showed combination 3 AND (the badger was still not infected at the following capture session when it was not captured OR the badger was infected at the following capture session when it was not captured)).

The likelihood of a given capture history can be expressed as a function of the probability of the epidemiological state at first capture (*π*), the probability of capture (*C*), the probability of transition from uninfected to infected states between two capture sessions (*T*), and the probabilities of obtaining the different combinations of diagnostic test results for each epidemiological state (*P*_*inf,k*_ and *P*_*uninf,k*_). The model was extended to account for potential variation in probabilities *π*, *C* and *T* according to the sex of the badger, year and season of the capture session. The association between the probability of capture and the infection status at the individual level was also examined. An interaction between sex and season was included in the capture probability to account for the possibility that the capture pattern for males may differ across seasons from that of females due to reproductive and breeding behaviours. Finally, because capture sessions were not implemented perfectly regularly, the model was extended to account for unequal time intervals between capture sessions^45^. Individual capture histories were right-censored after the last capture, so that all individual capture histories were conditional on the survival of the badger throughout the observation period. This was included because the objective of the study was to estimate the probability of an individual being infected at the time of its last (most recent) capture. Model parameters to be estimated are precisely defined in Supplementary Table S4, and a multinomial tree diagram of the probability structure of the model is presented in Fig. 5. The likelihood of an individual capture history can be obtained by summing the probability of all possible paths leading to that capture history. The likelihood of a given path can be obtained by multiplying the probabilities appearing along this path. The likelihood of the whole dataset can be obtained by multiplying the probability of all individual capture histories.

Parameter estimation was conducted using the maximum likelihood approach implemented in the programme E-SURGE version 1.8^45^. Further details of this procedure can be found in the Supplementary Information file. The most complex model was fitted to the data and non-significant variables were removed following a backward elimination procedure based on the Akaike Information Criterion adjusted for over-dispersion (QAIC), until the removal of each remaining variable increased the QAIC by more than 2 points^46^.

### Estimation of the true epidemiological state of an individual at the time of its last capture

Using the parameters estimated by the best fit multi-event capture-recapture model, we applied the generalised Viterbi algorithm^47^ to compute, for each badger, the probability *P*(*Inf*|*Hist*) of being infected at its most recent capture given its history of observations^48^. With *s*_*first*_ and *s*_*last*_ being respectively the first and the last capture sessions at which it was caught, *P*(*Inf*|*Hist*) was computed for each captured badger as follows:

1 $$P(Inf|Hist)=\,\frac{P(Inf{\cap }^{}Hist)}{P(Inf{\cap }^{}Hist)+P(Uninf{\cap }^{}Hist)}.$$

The event “*Uninf*” (the badger was uninfected at its most recent capture) corresponding to the single event “*the badger was uninfected at its first capture and never subsequently became infected*”, $P(Uninf\cap Hist)$ can be calculated as

2 $$P(Uninf\cap Hist)=P(Uninf)\ast P(Hist|Uninf)$$

with

3 $$P(Uninf)=(1-{\pi }_{{s}_{first},i})\prod _{s={s}_{first}}^{{s}_{last}-1}(1-{T}_{s,i})$$

and *P*(*Hist*|*Uninf*) expressed as a function of the probabilities of obtaining each test result combination given the individual is uninfected.

Calculating $P(Inf{\cap }^{}Hist)$ needs integrating over all possible state histories that could lead to the event “the badger was infected at the last session in which it was caught”, i.e. over all possible transitions from the uninfected to the infected state before the last session at which it was caught. These state histories include all events from “the badger was already infected at first capture” to “the badger became infected just before its last capture”. Therefore, it can be expressed as

4 $$\begin{array}{rcl}P(Inf\cap Hist) & = & P(already\,Inf\,at\,{s}_{first}\cap Hist)\\  &  & +P(was\,first\,Inf\,at\,({s}_{first}+1)\cap Hist)\\  &  & +\sum _{{s}_{inf}={s}_{first+2}}^{{s}_{last}}P(was\,first\,Inf\,at\,{s}_{inf}\cap Hist)\end{array}$$

with

5 $$P(already\,Inf\,at\,{s}_{first}\cap Hist)={\pi }_{{s}_{first}}\ast P(Hist|already\,Inf\,at\,{s}_{first}),$$

6 $$P(was\,first\,Inf\,at({s}_{first}+1)\cap Hist)=(1-{\pi }_{{s}_{first}})\ast {T}_{{s}_{first}}\ast P(Hist|was\,first\,Inf\,at\,({s}_{first}+1))$$

and

7 $$P(was\,first\,Inf\,at\,{s}_{inf}\cap Hist)=(1-{\pi }_{{s}_{first}})\ast [\prod _{s={s}_{first}}^{{s}_{inf}-2}(1-{T}_{s,i})]\ast {T}_{{s}_{inf}-1}\ast P(Hist|was\,first\,Inf\,at\,\,{s}_{inf}).$$

Using the information presented in Supplementary Table S3, it is straightforward to express *P*(*Hist*|*already Inf at s*_*first*_), *P*(*Hist*|*already Inf at* (*s*_*first*_+1)) and *P*(*Hist*|*was first Inf at s*_*inf*_) as functions of the probabilities of obtaining each test result combination given the epidemiological state of the individuals.

The end result was a numerical estimate of the probability of infection for each individual, ranging from zero (definitely uninfected) to 1.00 (definitely infected).

## Electronic supplementary material

Supplementary Information file

Supplementary Data file

## References

[1] Choquet R, Carrie C, Chambert T, Boulinieri T (2013). Estimating transitions between states using measurements with imperfect detection: application to serological data. Ecology.

[2] Daszak P, Cunningham AA, Hyatt AD (2000). Emerging infectious diseases of wildlife - threats to biodiversity and human health. Science.

[3] Jones KE (2008). Global trends in emerging infectious diseases. Nature.

[4] Graunt, J. Natural and political observations made upon the bills of mortality. London: John Martin (1662).

[5] Chao A, Tsay PK, Lin S-H, Shau W-Y, Chao D-Y (2001). The applications of capture-recapture models to epidemiological data. Statistics in Medicine.

[6] Laplace, S. P. Sur les naissances, les mariages et les morts. Histoire de l’Académie Royale des Sciences Année 1783. **693** (1786).

[7] Petersen C (1896). The yearly immigration of young plaice into the Limfjord from the German sea. Report of the Danish Biological Station.

[8] Hook EB, Regal RR (1995). Capture-recapture methods in epidemiology: methods and limitations. Epidemiologic Reviews.

[9] Vergne T, Vilas DR, Cameron VJ, Dufour AB, Grosbois V (2015). Capture-recapture approaches and the surveillance of livestock diseases: A review. Preventive Veterinary Medicine.

[10] Conn PB, Cooch EG (2009). Multistate capture–recapture analysis under imperfect state observation: an application to disease models. Journal of Applied Ecology.

[11] Lachish S, Jones M, McCallum H (2007). The impact of disease on the survival and population growth rate of the Tasmanian devil. Journal of Animal Ecology.

[12] Rossi S (2011). New insights on the management of wildlife diseases using multi-state recapture models: the case of classical swine fever in wild boar. PLoS One.

[13] Santoro S, Pacios I, Moreno S, Berto-Moran A, Rouco C (2014). Multi-event capture-recapture modeling of host-pathogen dynamics among European rabbit populations exposed to myxoma and Rabbit Hemorrhagic Disease Viruses: common and heterogeneous patterns. Veterinary Research.

[14] Jennelle CS, Cooch EG, Conroy MJ, Senar JC (2007). State-specific detection probabilities and disease prevalence. Ecol Appl.

[15] Lebreton JD, Pradel R (2002). Multistate recapture models: Modelling incomplete individual histories. Journal of Applied Statistics.

[16] Strain, S. A. J., McNair, J. & McDowell, S. W. J. Bovine tuberculosis: a review of diagnostic tests for *M. bovis* infection in cattle. Report: Agri-Food and Biosciences Institute. Available online: http://www.dardni.gov.uk/afbi-literature-review-tb-review-diagnostic-tests-cattle.pdf (accessed 22 March 2016) (2011).

[17] Strain, S. A. J., McNair, J. & McDowell, S. W. J. Bovine tuberculosis: a review of diagnostic tests for *M. bovis* infection in badgers. Report: Agri-Food and Biosciences Institute. Available online: http://www.dardni.gov.uk/afbi-literature-review-tb-review-diagnostic-tests-badgers.pdf (accessed 22 March 2016) (2011).

[18] Dohoo, I., W., M. & Stryhn, H. Screening and diagnostic tests. In: Veterinary Epidemiologic Research, 2nd edition. Charlottetown, Canada: VER Inc, p. 111 (2009).

[19] Drewe JA, Tomlinson AJ, Walker NJ, Delahay RJ (2010). Diagnostic accuracy and optimal use of three tests for tuberculosis in live badgers. PLoS One.

[20] Buzdugan SN, Chambers MA, Delahay RJ, Drewe JA (2016). Diagnosis of tuberculosis in groups of badgers: An exploration of the impact of trapping efficiency, infection prevalence and the use of multiple tests. Epidemiology and Infection.

[21] Cooch EG, Conn PB, Ellner SP, Dobson AP, Pollock KH (2012). Disease dynamics in wild populations: modeling and estimation: a review. Journal of Ornithology.

[22] Graham J (2013). Multi-state modelling reveals sex-dependent transmission, progression and severity of tuberculosis in wild badgers. Epidemiology and Infection.

[23] Pradel R (2005). Multievent: an extension of multistate capture-recapture models to uncertain states. Biometrics.

[24] Lebreton JD, Burnham KP, Clobert J, Anderson DR (1992). Modeling survival and testing biological hypotheses using marked animals: A unified approach with case studies. Ecological Monographs.

[25] Lebreton JD, Nichols J, Barker RJ, Pradel R, Spendelow JA (2009). Modeling individual animal histories with multistate capture–recapture models. Advances in Ecological Research.

[26] Delahay RJ (2013). Long-term temporal trends and estimated transmission rates for *Mycobacterium bovis* infection in an undisturbed high-density badger (*Meles meles*) population. Epidemiology and Infection.

[27] Delahay RJ, Langton S, Smith GC, Clifton-Hadley RS, Cheeseman CL (2000). The spatio-temporal distribution of *Mycobacterium bovis* (bovine tuberculosis) infection in a high-density badger population. Journal of Animal Ecology.

[28] Garnett BT, Delahay RJ, Roper TJ (2005). Ranging behaviour of European badgers (*Meles meles*) in relation to bovine tuberculosis (*Mycobacterium bovis*) infection. Applied Animal Behaviour Science.

[29] Garnett BT, Delahay RJ, Roper TJ (2002). Use of cattle farm resources by badgers (*Meles meles*) and risk of bovine tuberculosis (*Mycobacterium bovis*) transmission to cattle. Proceedings of the Royal Society B.

[30] Weber N (2013). Denning behaviour of the European badger (*Meles meles*) correlates with bovine tuberculosis infection status. Behavioral Ecology and Sociobiology.

[31] Woodroffe R (2006). Culling and cattle controls influence tuberculosis risk for badgers. Proceedings of the National Academy of Sciences.

[32] Derrick SC (2008). The safety of post-exposure vaccination of mice infected with *Mycobacterium tuberculosis*. Vaccine.

[33] Turner J (2000). Effective pre-exposure tuberculosis vaccines fail to protect when they are given in an immunotherapeutic mode. Infection and Immunity.

[34] Tuyttens FAM (1999). Differences in trappability of European badgers *Meles meles* in three populations in England. Journal of Applied Ecology.

[35] Kruuk H, Parish T (1985). Food, food availability and weight of badgers (*Meles meles*) in relation to agricultural changes. Journal of Applied Ecology.

[36] Tolhurst BA, Delahay RJ, Walker NJ, Ward AI, Roper TJ (2009). Behaviour of badgers (*Meles meles*) in farm buildings: Opportunities for the transmission of *Mycobacterium bovis* to cattle?. Applied Animal Behaviour Science.

[37] Page RJC, Ross J, Langton SD (1994). Seasonality of reproduction in the European badger *Meles meles* in south-west England. Journal of Zoology.

[38] King HC (2015). Performance of a non-invasive test for detecting *Mycobacterium bovis* shedding in European badger (*Meles meles*) populations. Journal of Clinical Microbiology.

[39] Peeling RW, Mabey D (2010). Point-of-care tests for diagnosing infections in the developing world. Clinical Microbiology and Infection.

[40] Cheeseman CL, Harris S (1982). Methods of marking badgers (*Meles meles*). Journal of Zoology.

[41] Goodger J (1994). Serodiagnosis of *Mycobacterium bovis* infection in badgers: development of an indirect ELISA using a 25 kDa antigen. Veterinary Record.

[42] Greenwald R (2003). Improved serodetection of *Mycobacterium bovis* infection in badgers (*Meles meles*) using multiantigen test formats. Diagnostic Microbiology and Infectious Disease.

[43] Dalley D (2008). Development and evaluation of a gamma-interferon assay for tuberculosis in badgers (*Meles meles*). Tuberculosis (Edinb).

[44] Clifton-Hadley RS, Wilesmith JW, Stuart FA (1993). *Mycobacterium bovis* in the European badger (*Meles meles*): epidemiological findings in tuberculous badgers from a naturally infected population. Epidemiology and Infection.

[45] Choquet, R., Rouan, L. & Pradel, R. Programme E-SURGE: A software application for fitting multievent models. In: Modeling Demographic Processes in Marked Populations (Eds D. L. Thomson, E. G. Cooch and M. J. Conroy) Springer, New York, USA. (2009).

[46] Burnham, K. & Anderson, D. Mo0del Selection and Multimodel Inference: a Practical Information-Theoretic Approach. Springer, New York, USA (2002).

[47] Foreman LA (1992). Generalisation of the Viterbi algorithm. IMA Journal of Management Mathematics.

[48] Rouan, L., Gaillard, J. M., Guédon, Y. & Pradel, R. Estimation of lifetime reproductive success when reproductive status cannot always be assessed. In: David L. Thomson, Evan G. Cooch and Michael J. Conroy (Eds) Modeling Demographic Processes in Marked Populations. Springer Publishing, pp 867–879 (2009).

