# Supplementary material for: Inference of the infection status of individuals using longitudinal testing data from cryptic populations: Towards a probabilistic approach to diagnosis
Source: Sci Rep. 2017 Apr 19;7:1111. doi: 10.1038/s41598-017-00806-4 (PMC5430431; doi:10.1038/s41598-017-00806-4)
Supplement: Supplementary file 1 — Supplementary Information file [file 41598_2017_806_MOESM1_ESM.pdf]

Supplementary information

Inference of the infection status of individuals using longitudinal testing data from cryptic populations: Towards a probabilistic approach to diagnosis

Svetlana N. Buzdugan[a#], Timothée Vergne[a#], Vladimir Grosbois[b], Richard J. Delahay[c] and Julian A. Drewe[a]*

[a] *Royal Veterinary College, London, UK*
[b] *Centre de Coopération Internationale en Recherche Agronomique pour le Développement, Montpellier, France*
[c] *National Wildlife Management Centre, Animal and Plant Health Agency, Woodchester Park, Gloucestershire, UK*

#These authors contributed equally to this work

*Corresponding author: Julian A Drewe
Veterinary Epidemiology, Economics and Public Health Group
Royal Veterinary College
Hawkshead Lane
North Mymms
Herts AL9 7TA
UK
email: jdrewe@rvc.ac.uk
Telephone: +44 (0) 1707 666051

Contents

Title	Contents	Page
E-Surge methods	Description of the use of E-Surge software for fitting the model described in the paper	2
Supplementary Table S1	Distribution of the probability of being infected at first capture between 2007 and 2012 for badgers at Woodchester Park	3
Supplementary Table S2	The frequency of badger capture by season for 541 badgers (247 male and 294 female) from July 2006 to October 2013 at Woodchester Park	4
Supplementary Table S3	Probability of observing each combination of diagnostic test results given the epidemiological state (uninfected or infected) expressed as a function of the sensitivity (Se) and specificity (Sp) of each diagnostic test	5
Supplementary Table S4	Definitions of parameters used in the multi-event capture-recapture model	6

E-Surge methods

The following notes outline how to use the E-Surge software for fitting the model described in the paper.

The model was fitted to the data using the software E-Surge. This free software is dedicated to the statistical modelling of data resulting from longitudinal individual monitoring protocols where each individual is classified into one of a number of categories at each capture. These categories correspond to observations and/or measurements that reflect, not necessarily perfectly, the value of a state variable that can change over the monitoring period within monitored individuals

The first step in using E-Surge is to download the data (in the form of capture histories) and specify their characteristics (number of time intervals, length of the time intervals, number of categories into which individuals can be classified upon capture, number of possible individual states, number of distinct groups of individuals).

The second step is to specify the structure of the model used to depict the biological and observation processes that gave rise to the data. The model structure includes the specification of the parameters that govern the transitions among individual states and of the parameters that allow linking the categories characterizing individuals at capture to the different possible individual states.

The last step is to build the model's design matrix. The design matrix reflects how each of the parameters specified at the previous step varies (e.g. over time, between groups of individuals, etc.…).

Finally E-Surge uses sophisticated likelihood maximization algorithms to fit the specified model to the data and produces detailed output

For an in depth description of E-Surge we refer the reader to the paper by Choquet, Rouan & Pradel (2009) that introduces the program and to E-Surge user's manual (Choquet and Nogue 2011).

References

Choquet, R., & Nogue, E. (2011). E-SURGE 1.8 user's manual. English edition. This manual can be downloaded at http://www.cefe.cnrs.fr/fr/ressources/logiciels/34-french/recherche/bc/bbp/264-logiciels

Choquet, R, Rouan, L., and Pradel, R (2009) Program E-SURGE: a software application for fitting multievent models. In: Modelling demographic processes in marked populations. Springer US. pp. 845-865.

Supplementary Table S1: Distribution of the probability of being infected at first capture between 2007 and 2012 for badgers at Woodchester Park.

Year	Probability of being infected at first capture	95% confidence interval
2007	0.06	0.02, 0.18
2008	0.33	0.22, 0.47
2009	0.25	0.16, 0.36
2010	0.21	0.10, 0.38
2011	0.40	0.20, 0.64
2012	0.11	0.04, 0.29

Supplementary Table S2. The frequency of badger capture by season for 541 badgers (247 male and 294 female) from July 2006 to October 2013 at Woodchester Park. Badgers were caught multiple times (median: 3 times per badger, range: 1 to 18 times) giving a total of 2,022 sampling events during the study period.

**Season**	**Number (%) of captures***		
	**Males**	**Females**	**All**
**Winter** (2 December to 30 March)	151 (16)	86 (8)	237 (12)
**Spring** (1 April to 30 June)	259 (27)	358 (34)	617 (31)
**Summer** (1 July to 2 October)	327 (34)	385 (37)	712 (35)
**Autumn** (3 October to 1 December)	232 (24)	224 (21)	456 (23)
Total	969 (100)	1053 (100)	2022 (100)

*Any capture sessions that overlapped two seasons were allocated to the season where most captures from that session took place. As a result, four seasons during the 7.5-year study period contained no capture session (summer 2009, winter 2010, spring 2011, autumn 2011) whereas the seasons adjoining these each contained two capture sessions.

Supplementary Table S3: Probability of observing each combination of diagnostic test results given the epidemiological state (uninfected or infected) expressed as a function of the sensitivity (Se) and specificity (Sp) of each diagnostic test.

Test result combination	Test 1 (StatPak)	Test 2 (IFNγ)	Test 3 (Culture)	Probability of the combination of test results	
				if uninfected	if infected
1	-	-	-	$Sp_1*Sp_2*Sp_3$	$(1-Se_1)*(1-Se_2)*(1-Se_3)$
2	+	-	-	$(1-Sp_1)*Sp_2*Sp_3$	$Se_1*(1-Se_2)*(1-Se_3)$
3	-	+	-	$Sp_1*(1-Sp_2)*Sp_3$	$(1-Se_1)*Se_2*(1-Se_3)$
4	-	-	+	$Sp_1*Sp_2*(1-Sp_3)$	$(1-Se_1)*(1-Se_2)*Se_3$
5	+	+	-	$(1-Sp_1)*(1-Sp_2)*Sp_3$	$Se_1*Se_2*(1-Se_3)$
6	+	-	+	$(1-Sp_1)*Sp_2*(1-Sp_3)$	$Se_1*(1-Se_2)*Se_3$
7	-	+	+	$Sp_1*(1-Sp_2)*(1-Sp_3)$	$(1-Se_1)*Se_2*Se_3$
8	+	+	+	$(1-Sp_1)*(1-Sp_2)*(1-Sp_3)$	$Se_1*Se_2*Se_3$

+ = positive test result; - = negative test result

Supplementary Table S4: Definitions of parameters used in the multi-event capture-recapture model.

Parameter	Definition
$\pi_{s,i}$	Probability that a badger of sex *i* is infected at its first encounter at capture session *s*
$T_{s,i}$	Probability that a badger of sex *i* that is uninfected at capture session *s* will be infected at the capture session *s*+1
$C_{s,i,inf}$	Probability that an infected badger of sex *i* is captured at capture session *s*
$C_{s,i,sus}$	Probability that an uninfected badger of sex *i* is captured at capture session *s*
$P_{inf, k}$	Probability of observing the combination of diagnostic test result *k* given the true epidemiological status is infected
$P_{uninf, k}$	Probability of observing the combination of diagnostic test result *k* given the true epidemiological status is uninfected
